# What Motivates Smokers to Switch to ENDS? A Qualitative Study of Perceptions and Use

**DOI:** 10.3390/ijerph17238865

**Published:** 2020-11-28

**Authors:** Abigail T. Evans, Katherine C. Henderson, Anna Geier, Scott R. Weaver, Claire Adams Spears, David L. Ashley, Meredith Fritz, Lisa John, Terry F. Pechacek

**Affiliations:** 1 Battelle Memorial Institute, Baltimore, MD 21209, USA; geier@battelle.org (A.G.); fritz@battelle.org (M.F.); johnl@battelle.org (L.J.); 2 School of Public Health, Georgia State University, Atlanta, GA 30303, USA; srweaver@gsu.edu (S.R.W.); cspears@gsu.edu (C.A.S.); dashley4@gsu.edu (D.L.A.); tpechacek@gsu.edu (T.F.P.)

**Keywords:** electronic cigarette, decision making, motivation, focus groups, dual use

## Abstract

Switching completely from cigarettes to electronic nicotine delivery systems (ENDS) may reduce health risks for addicted smokers. This paper provides information about perceptions and other factors that may influence smokers’ ENDS use and substitution for cigarettes. We conducted 12 online focus groups (*N* = 61) among smokers who had never tried using ENDS (Never Users, *N* = 11), currently used both cigarettes and ENDS (Dual Users, *N* = 21), used but discontinued ENDS (Rejectors, *N* = 14), and switched completely to ENDS use (Switchers, *N* = 15). Thematic analysis was used to interpret the transcripts. Participants described initial interest in trying ENDS in hopes of quitting smoking and because of convenience (i.e., due to rules, regulations, or social norms). Risk perceptions and higher prices relative to cigarettes were reported as disadvantages of ENDS that discouraged initiation. Dual Users and Rejectors reported product problems (e.g., products breaking) and dissatisfaction (i.e., inability to satisfy cravings for cigarettes) as factors that lowered their substitutability for cigarettes or led to discontinuing ENDS use. Switchers indicated that satisfaction, lack of product problems, and perceived safety facilitated successfully switching from cigarette smoking to exclusive ENDS use. However, Switchers reported trying many products before they found ones that satisfied their needs. We recommend that policymakers consider the potential impact of tobacco control policies on smokers’ motivation and ability to switch completely from cigarettes to ENDS.

## 1. Introduction

Current scientific evidence suggests that, although electronic nicotine delivery systems (ENDS) are not harmless, completely switching from combustible cigarettes to ENDS offers a pathway to significant harm reduction for addicted smokers [1,2,3]. However, surveillance data suggest that most smokers who try ENDS do not switch completely from cigarettes to ENDS [4,5]. To successfully switch from cigarettes to ENDS, smokers must make a series of decisions including the decisions to try ENDS, to continue using ENDS, and to stop smoking cigarettes while continuing to use ENDS. The current research aims to build our understanding of how smokers’ perceptions of and experiences with ENDS influence these decisions, which could have implications for tobacco control policy.

Smokers who have tried ENDS often report that their initial decision to do so was driven by their desire to quit or to cut back on smoking cigarettes [6,7]. Other reasons, such as believing that ENDS are less harmful than cigarettes, curiosity about e-cigarettes, interest in flavors, and greater social acceptability of ENDS compared to cigarettes have also been reported [7,8,9,10]. Given the potential health benefits of switching completely from cigarettes to ENDS, it is also important to understand the reasons that smokers decide not to try ENDS. Although most smokers report that they would like to quit smoking, nearly 40% of smokers have not yet tried ENDS [11]. Common reasons smokers gave for not trying ENDS included not wanting to become addicted to ENDS and not believing that ENDS could help them quit smoking [11]. Between 2017 and 2018, the proportion of adults who believed ENDS were less harmful than cigarettes decreased and the proportion who believed they were more harmful than cigarettes increased [12]. Reports of e-cigarette or vaping associated lung injury [13] may have further reduced smokers’ willingness to try ENDS.

The factors that influence a smoker’s decision to try or not try ENDS may not be the same as the factors that influence how they consume ENDS in relation to their smoking or whether they discontinue use. For example, one nationally representative survey found that over half of the ENDS users surveyed cited consideration for others and convenience as reasons for using ENDS [14]. However, these reasons have not been widely identified as a reason for initially trying ENDS. Evidence that ENDS can help smokers quit, particularly when used under real-world use conditions, remains mixed [15,16,17,18]. The reasons ENDS have not been shown to consistently help smokers quit are likely complex. For example, some ENDS products appear to be more effective as smoking cessation aids than others [6,19,20]. Some ENDS users report stopping ENDS use because their product did not feel like smoking or did not have a satisfying taste [21]. Smokers who have successfully switched to ENDS advise smokers trying to make the switch that they need to find the product and nicotine combination that “works for them” and that they should be open to continuing to smoke while vaping in the beginning [22]. However, the factors that influence dual use, discontinuation of ENDS with continued smoking, and switching completely to ENDS remain poorly understood. Thus, the current research sought to explore how perceptions, experiences, and policy-relevant variables, such as the availability of flavors or smokers’ ability to use ENDS in places where smoking is not acceptable affect smokers’ decisions to try ENDS, continue to dual use, switch from cigarettes to ENDS or relapse to exclusive cigarette smoking. In other words:

1. Why do smokers decide to try using ENDS or not try using ENDS?
2. What factors drive dual users of cigarettes and ENDS to use both products rather than switching completely to ENDS?
3. What factors drive smokers who have initiated ENDS use to discontinue and return to smoking cigarettes?
4. What factors facilitate a successful transition from cigarette smoking to exclusive ENDS use?

To accomplish this, we conducted focus groups with a cross section of smokers and former smokers with different patterns of ENDS use/non-use.

## 2. Materials and Methods

Twelve focus groups were conducted in three U.S. cities. In each city, a group was conducted for smokers who had never used ENDS (Never Users), dual-users of both cigarettes and ENDS (Dual Users), smokers who initiated ENDS but discontinued use (Rejectors), and former smokers who had switched exclusively to ENDS (Switchers). Three focus groups were conducted for each subgroup based on the recommendation to conduct three groups on a given topic to analyze information across groups [23,24].

### 2.1. Recruitment and Eligibility

Smokers were recruited for in-person focus groups from three U.S. cities (Atlanta, GA, Baltimore, MD, and St. Louis, MO) in February–March 2020 via targeted Facebook and Craigslist advertisements directing individuals to an online screener survey. A research coordinator contacted by telephone the respondents who appeared eligible to confirm eligibility and subgroup placement. Eligibility criteria for the study included having smoked more than 100 lifetime cigarettes and being at least 21 years of age. Eligibility for subgroups was determined by current and past 30-day cigarette and ENDS use patterns. Never Users reported that they currently smoked cigarettes every day or some days and had never used an electronic vapor product that contained nicotine (even one or two times) at the time of screening. Dual Users currently smoked cigarettes every day or some days and also used an electronic vapor product that they owned every day or some days at the time of screening. Rejectors reported that they currently smoked cigarettes every day or some days, had previously used an electronic vapor product that they owned, and had not used an electronic vapor product in the past 30 days at the time of screening. Switchers reported that they had smoked at least 100 cigarettes in their lifetime, but did not currently smoke cigarettes and currently used an electronic vapor product that they owned every day or some days. Focus groups were initially scheduled to take place in person, but were shifted to an online platform due to government recommendations related to COVID-19.

### 2.2. Procedure

Focus groups were conducted from 13 March 2020 through 26 March 2020 and lasted between 90 and 120 min each. Participants were scheduled for groups based on their cigarette and ENDS use status determined by their responses to the online screener and follow-up screening call. At the beginning of each group, participants provided informed consent. Participants then completed a questionnaire that assessed demographics (age, race, ethnicity, educational attainment, and sexual orientation) and asked further details of their past and current ENDS use behavior (e.g., frequency of use, types of ENDS products used).

The focus groups were led by a moderator who guided participants through a semi-structured script specific to each subgroup. The same moderator led all groups. Questions in the moderator guide covered preferences for specific product characteristics; cognitive, affective, and contextual factors affecting decisions to use ENDS vs. combustible cigarettes; barriers to quitting smoking; absolute and relative risk perceptions; and reasons for rejecting ENDS or not switching completely.

The Adobe Connect online platform was used to conduct all focus groups, with technical assistance from Focus Vision. A debriefing document and resources were available for participants to download after the groups concluded. Participants were compensated $90. All participants gave their informed consent for inclusion before they participated in the study. The study was conducted in accordance with the Declaration of Helsinki, and the protocol was approved by the Georgia State University Institutional Review Board (IRB Number: H19670, Reference Number: 358678) on 28 January 2020.

### 2.3. Qualitative Coding and Analysis

All focus groups were recorded and transcribed verbatim. Transcripts were coded using NVivo v.11 (QSR International, Melbourne, Australia) [25]. Both inductive and deductive approaches were used to code the qualitative data. A preliminary set of codes was developed based on the moderator guide and additional codes were added to capture topics that emerged during group discussion. The codebook was used by a team of four independent coders who all coded subsets of the data, after which the team convened to discuss areas of coding disagreement and to refine and update the codebook. The revised codebook was then used to code subsequent sets of data. These subsets were used to establish inter-rater reliability, which was considered sufficient based on kappa ≥0.80 [26]. After the team reached reliability, a single coder coded each transcript. A second coder double coded approximately 20% of each transcript to ensure continued reliability. This double coding was performed using a round-robin scheme, such that each coder’s transcripts were double coded by each other member of the coding team. The coders discussed disagreements as a group until consensus was achieved and coding was adjusted accordingly. Coding was used to identify major themes within and between subgroups of smokers.

## 3. Results

### 3.1. Participant Sociodemographics

Table 1 summarizes the demographic variables by ENDS use status. A total of 61 smokers and former smokers participated in the focus groups including 11 Never Users, 14 Rejectors, 21 Dual Users, and 15 Switchers. Overall, the sample was 69% female, 85.2% heterosexual, 47.5% Black or African American, 44.3% had a university degree or higher, and the average age was 45.9 years (SD = 11.5 years). Although the demographic compositions of the Never Users, Rejectors, and Dual Users groups were similar to one another, the Switchers group included approximately half females (53.3% female) and no Black or African American participants.

### 3.2. Participant Electronic Nicotine Delivery Systems (ENDS) Use Patterns

Participants were scheduled for groups based on their self-reported cigarette and ENDS use status determined during the screening process. During the screening process, participants reported limited information on their ownership and current and last use of ENDS. However, they were also asked further details about their level and frequency of ENDS use in a survey administered immediately prior to the focus group interview (“pre-focus group survey”). Participants may have interpreted these questions differently, which could have led to different results in the screening compared to the pre-focus group survey. Moreover, the screening call was administered 2–3 weeks prior to the focus group interview and pre-focus group survey which could have resulted in a change in use status between the screening and the focus group interview.

Table 2 summarizes participants’ ENDS use patterns and product characteristics by group as assessed by a questionnaire each participant completed at the beginning of the focus group interview. In the pre-focus group survey, all Switchers (100%) and most Dual Users (61.9%) reported having used ENDS in the past 7 days (all reported using ENDS in the past 30 days), while most Rejectors (92.9%) had last used ENDS more than 30 days ago. Although Never Users indicated that they had never used an ENDS product that contained nicotine (even one or two times) during initial screening, two (18.2%) reported that they had used an ENDS product six months ago or more in the pre-focus group survey. However, they indicated that they had only used an ENDS product one time, which suggests experimentation with products that they likely did not own. Most Switchers reported using ENDS every day (93.3%), most Dual Users reported using ENDS some days (52.4%), and most Rejectors reported not currently using ENDS (78.6%); Although three Rejectors reported that they currently used ENDS “some days” only one Rejector user reported having used an ENDS product in the past 30 days or less.

The moderator’s guide indicated that all focus groups should begin with a conversation about participants’ experience using ENDS. These conversations lent context to participants’ experiences with smoking cigarettes and using ENDS. Although two Never Users indicated that they had tried ENDS once on the pre-focus group survey (Table 2), they did not self-identify as ever-users of ENDS or as owning an ENDS device during discussion, suggesting casual experimentation rather than use initiation. Rejectors indicated that they had previously owned a device and used ENDS regularly, but did not currently use ENDS. Although one Rejector indicated that they had used ENDS in the past 7 days on the pre-focus group survey (Table 2), they did not self-identify as a current ENDS user during this conversation. Dual Users all self-identified as current ENDS users and cigarette smokers, indicating ongoing use of both products. However, there was variability in how frequently they used each product with some reporting more lifetime and recent use of ENDS than others (Table 2). Switchers self-identified as having switched from smoking cigarettes to using ENDS during the discussion. Most Switchers discussed either having used cigarettes and ENDS simultaneously before switching completely to ENDS or stopping cigarette use only once they started using ENDS.

When asked about the product they use most often in the pre-focus group survey, most ENDS users (Rejectors, Dual Users, and Switchers) reported using a rechargeable product (68.9%). Whereas most Rejectors and Dual Users reported using pod systems (57.1% and 61.9%, respectively), most Switchers reported using tank systems (80%). The use of refillable products was common among Rejectors and Dual Users (50% and 42.9%, respectively) and very common among Switchers (86.7%).

### 3.3. Emerging Themes

When examining the data, several themes emerged as influencing participants’ decisions to try, not try, continue using, or discontinue using ENDS. These themes were created using both inductive and deductive analysis approaches. Concepts and experiences were grouped together where consistently linked in participants’ discussions. Each theme played a different role for each smoker group. There were no major differences in themes by city within each smoker group. Due to the high quality of discussions, we were able to reach thematic saturation. The main themes, as identified in our analysis, are listed in Table 3 below.

Table 4 summarizes the main findings from our analysis by research question and provides illustrative quotes. The four research questions and main findings are explored further in subsequent sections.

### 3.4. Research Question 1: Why Do Smokers Decide to Try Using ENDS or Not to Try Using ENDS?

Smokers’ willingness to try ENDS was influenced by four main factors: smoking cessation beliefs, convenience, risk perceptions, and cost. Many participants consciously debated the advantages and drawbacks of ENDS use vs. cigarette smoking. The weight they placed on each factor appeared to play a role in smokers’ decision-making process in initiating and continuing ENDS use.

#### 3.4.1. All Groups Were Initially Interested in ENDS Because of Smoking Cessation Beliefs

Many participants from all ENDS-using groups indicated that they began using ENDS because they hoped ENDS would help them quit smoking. Switchers and Dual Users also mentioned the perceived potential for smoking cessation was a motivating factor for having tried a wider variety of ENDS products. Although not all participants wanted to quit smoking, most indicated that if they were ready to quit, ENDS’ perceived ability to help with this goal would make them even more appealing.

*Female, Rejector: “My purpose for trying them was actually—I thought it would curb me from smoking cigarettes. I decided I was going to try to quit smoking. I thought that would aid in stopping […].”*

#### 3.4.2. Perceived Convenience Made Trying ENDS Appealing for All Groups

Participants from all groups indicated that they first wanted to try ENDS because of their perceived convenience in certain places or social situations. Specifically, participants mentioned first trying ENDS, or wanting to try ENDS, because of the perceived acceptability of vaping indoors and around non-smokers.

*Female, Never User: “Even—I guess if I could just have [an ENDS device] in my hand, to just—say if you’re at a conference or something and it’s five, six, seven hours, something like that, and I’ve got it in my hand, yeah, that would probably deter me from wanting to go outside and smoke.”*

Participants often cited the perceived lack of cigarette smell on their clothes, hands, car, and home when using ENDS as motivating them to try ENDS. They perceived the smell of ENDS to be more discreet and socially acceptable to use, especially around children and non-smokers.

*Female, Dual User: “As soon as I walk in the house, I know I smell and I pick up my two-year old granddaughter, and I know it’s on her. I don’t like that, because none of them smoke. I’d rather go ahead and use an electronic cigarette, that way I know it’s OK, and I’m not smelling, and reeking of cigarettes.”*

#### 3.4.3. Risk Perceptions May Deter or Encourage Trying or Re-Trying ENDS

Concerns about perceived health risks deterred some Never Users and Rejectors from trying or re-trying ENDS products. For example, participants mentioned concerns about products exploding, lung problems, popcorn lung, and the recent hospitalization of young people. For Never Users, these concerns prevented them from trying ENDS completely, and for Rejectors, these concerns made them hesitant to try ENDS initially or prevented them from trying new ENDS products. These more immediate health risks, in comparison to the long-term health risks of smoking cigarettes, were cited by some participants as preventing them from trying or re-trying ENDS.

*Male, Never User: “I’ve never tried it, based on what I’ve read. It had such an impact on the lungs and over the years, the years that I’d smoke—it was always that fear—I knew that smoking would damage my lungs, and my fear was that the vaping would damage it even more, so therefore, I never started it. Then on top of that, you added the—the news, in turn helped me not to even think. It scared me to death, so therefore I thought it wasn’t in my best interest.”*

Others were concerned by the lack of long-term research around ENDS and the unknowns about the health effects. Most Dual Users and Switchers were not deterred by this, indicating that they weighed the health risks of cigarettes as greater than the unknowns of ENDS when deciding to initially try ENDS. Some even asserted that ENDS have lower risks than cigarettes but did not ascribe this to the reasons they initiated ENDS.

*Male, Dual User: “One of the risks of using vapes is that it does not have the decades’ worth of studies behind it, like you would with cigarettes, so that to me is one of the risks—not knowing for sure, what it is actually doing to me, but in my mind, I automatically always assume that vaping is better than smoking cigarettes.”*

Many Never Users asserted that ENDS would need to be proven to be lower risk or healthier than cigarettes before they would begin using them.

*Female, Never User: “To me, I think it’s a big thing. Those two things—the data and then giving up one just to smoke another, those both to me are huge. I would want to see the data and know it’s safer than smoking a regular cigarette before I tried it.”*

#### 3.4.4. Cost Relative to Cigarettes Deterred Some from Trying or Re-Trying ENDS and Not Others

For some Never Users and Rejectors, the perceived high price of ENDS compared to cigarettes negatively impacted their willingness to try or re-try ENDS. Many Never Users indicated that ENDS would need to be less expensive than cigarettes to motivate them to try ENDS.

*Female, Never User: “No, I won’t buy it at all, because first of all, like she said, the money—I barely have enough money for my cigarettes, so I know I won’t have enough to buy that.”*

Overall, participants from all groups acknowledged the potentially high price of ENDS use as a possible disadvantage. Many ENDS users anecdotally mentioned that they had spent more than they intended to on ENDS products.

*Male, Switcher: “Sometimes I’ve had to buy an $80 unit, as far as the batteries, and all the other gear with it, you can drop $100 easy when you’re ready, and that will always be one of my biggest dislikes about vaping.”*

However, some Switchers and Dual Users rationalized that the wide variety of products on the market can accommodate all budgets and that they saved money in the long run. The perception of long-term savings, often motivated Switchers and Dual Users to continue trying new ENDS products. They asserted that once they found the right product, the low cost of ENDS use was an advantage.

*Female, Dual User: “I prefer the big [ENDS devices]—the big tank kind, that’s refillable, and it’s also better because money-wise, you buy the juice, you know how much juice you’re using. It really is—it’s a little bit of an investment to get started, but once you’re using it, it’s inexpensive.”*

### 3.5. Research Question 2: What Factors Drive Dual Users of Cigarettes and ENDS to Use Both Products Rather than Switching Completely to ENDS?

Although Dual Users continued to use ENDS because of their convenience, flavor variety, and potential to aid in smoking cessation, ENDS were unable to completely fulfill certain needs. Smokers described that their inability to switch completely to ENDS was influenced by two main factors: lack of craving satisfaction and product problems.

#### 3.5.1. Dual Users Were Motivated to Continue ENDS Use Because of Convenience

The convenience of ENDS use in certain places and social situations was appealing to all user groups but was particularly important to Dual Users. Dual Users reported switching from cigarettes to ENDS use throughout the day to manage smoke-free areas and social stigma.

*Male, Dual User: “I’m just going to use both of them [cigarettes and ENDS] because they have their time and their place.”*

*Male, Dual User: “I just think if you’re around people that don’t smoke, [it’s] considerate to not smoke in their house or around them because they don’t want to smell like smoke. I’ll just go outside and use the vape because it doesn’t leave any smell, and it doesn’t leave any smoke.”*

#### 3.5.2. Dual Users Found ENDS Flavors Appealing

Dual Users found ENDS flavors appealing and reported that they enjoyed having the option of trying a variety of flavors. Several people indicated that having different flavor options was desirable and helped prevent boredom with just one flavor. However, the flavors participants preferred varied. Some users indicated that they wanted flavors that mimicked their cigarette flavor and others said that they wanted candy and fruit flavors.

*Male, Dual User: “I like the options. I like to have different flavors, even different brands and everything. As opposed to cigarettes, you’ve just got cigarettes.”*

#### 3.5.3. Dual Users Hoped for Eventual Smoking Cessation

While Dual Users often initially tried ENDS with the belief that ENDS would help them quit smoking cigarettes, maintenance of this hope over time varied. Some became discouraged and disappointed that their ENDS product did not help them quit smoking and continued use for the other perceived benefits. Others indicated that they keep using ENDS and trying new products in the hopes that they would find a way to make ENDS work as a cessation aid.

*Female, Dual User: “Yeah. I was using [my vape] to quit and it actually helped me quit. I don’t know why I picked back up smoking again, but I guess because I just like that, too, so I’m back on [smoking] again […] But, I think I’m going back to the vape.”*

*Female, Dual User: “She asked why [do] we continue to smoke and vape. I was just going to say I thought vaping was going to help me not smoke cigarettes, but for me, I didn’t really see a reduction and I didn’t really see that it’s taken the place of cigarettes for me. Like I said, I was probably, didn’t give it enough time or just didn’t have the right product. But if it could help me not smoke and cut back, that would be appealing to me.”*

#### 3.5.4. ENDS Were Not Satisfying Enough to Support Switching for Dual Users

While convenience, flavors and smoking cessation motivated the continued use of ENDS among Dual Users, many stated that ENDS did not provide the same satisfaction as cigarettes. This emerged as the major reason Dual Users were unable to switch completely. Some Dual Users reported that ENDS were able to “take the edge off” when cigarette smoking was not possible; however, they were unable to fully satisfy their cravings. Dual Users also indicated that ENDS use was unsuitable for certain smoking rituals (e.g., having cigarettes with their morning coffee or while drinking alcohol).

*Female, Dual User: “When you vape, you don’t feel that quite sensation like you do when you smoke a cigarette in the morning.”*

*Male, Dual User: “So, I can use the vape products anytime, anywhere, really like I said earlier, it’s more socially acceptable. As for the cigarettes, I feel like if it wasn’t for my desperation after eating, or first thing in the morning after getting up, those are the two times that I have to have a cigarette, but throughout the day, I would prefer to vape, no matter where I am.”*

#### 3.5.5. Product Problems Prevented Dual Users from Switching Completely to ENDS

Dual Users also referenced experiencing product problems and indicated that they could not find an ENDS product that was "right" for them. Participants’ definitions of the “right” product varied and several Dual Users indicated that they tried different products in search of the right one. Although some wanted ENDS use to mimic certain smoking experiences, such as the size, weight, taste, and throat hit of cigarettes, others wanted to stay away from features that reminded them of cigarettes to avoid triggering cravings. The absence of these desired characteristics and experiences with the ENDS product they used deterred switching to exclusive ENDS use for many.

*Male, Dual User: "I haven’t found my ideal one yet. I think I’m still looking. I just haven’t found one that’s the right size or the right look. I think a lot of times with cigarettes, you pretty quickly find out which cigarettes you like and which ones you don’t. […] I just don’t think, for the vapes, I’ve really found it yet.”*

### 3.6. Research Question 3: What Factors Drive Smokers Who Have Initiated ENDS Use to Discontinue and Return to Smoking Cigarettes?

Discontinuation of ENDS use among Rejectors was influenced by four main factors: lack of satisfaction, product problems, cost, and risk perceptions. Unlike Dual Users who continued to use ENDS despite their issues, for Rejectors, these factors outweighed and often negated the potential for ENDS to serve as a smoking cessation aid or smoke-free policy avoidance strategy. When asked about flavors, Rejectors also expressed a particular disinterest that made continuing ENDS use less appealing.

#### 3.6.1. Rejectors Did Not Find ENDS Satisfying Enough

Rejectors often cited the inability of ENDS to satisfy their cravings as a major reason for discontinuing use. Unlike Dual Users, Rejectors indicated that, when compared to cigarettes, ENDS were inadequate because they did not provide the same enjoyment, satisfaction, or head rush. Many said that ENDS use was not similar enough to the “real thing,” indicating their dissatisfaction with ENDS’ inability to replicate the cigarette smoking experience.

*Female, Rejector: “I tried it. It wasn’t the same as a real cigarette, so I didn’t stick with it. I didn’t get the same enjoyment out of it.”*

*Female, Rejector: “Just give me my regular old tobacco. The taste was often funny to me. It wasn’t like smoking a cigarette, and I enjoy smoking a cigarette and I didn’t get any enjoyment. I tried it thinking it would help me back off of real cigarettes, but no.”*

When ENDS did not meet their satisfaction expectations, several Rejectors stopped using ENDS to try to quit smoking cigarettes. Some reported using ENDS mostly for the purpose of completely replacing their cigarettes, and when they felt this was not possible, they ceased use.

*Female, Rejector: “I never completely gave up regular cigarettes. I always just tried to, I guess, [use ENDS] in between, but I still smoke cigarettes. I tried [ENDS] since I thought it would help me quit […] I just never got the enjoyment out of it or satisfaction like I do from a cigarette.”*

*Male, Rejector: “I want to get off cigarettes. I really want to get off cigarettes. And during the time that I was not smoking [but] vaping. I was trying to replace cigarettes with vaping. And that didn’t work out for me at all. […] [I] got off vaping and went back to cigarettes. I don’t want to say absolutely there’s no chance that I would never do it [vape] again. But right now, it doesn’t look likely.”*

#### 3.6.2. Rejectors Were Frustrated by ENDS Product Problems

Rejectors reported abandoning ENDS because they experienced frustration with their ENDS device, saying that it was not the "right" product. However, they reported trying only a few products before discontinuing ENDS use. Rejectors indicated that the “right” product would be one that feels and tastes like their cigarettes, does not break easily, and does not need to charge frequently. Although the specific characteristics mentioned by each Rejector varied, overall, they indicated that ENDS should be as easy to use as cigarettes and should replicate the smoking experience.

*Male, Rejector: “And I really guess I quit using it mostly because I broke it. There was a glass tube and I remember breaking the thing. And I refused to go buy another one.”*

*Female, Rejector: “If they had one that when you held it, it felt like a cigarette. I don’t know how to explain it, but the ones they make now, it’s just so thick and heavy that you can’t hold it the same way.”*

#### 3.6.3. Rejectors Perceived ENDS as More Expensive than Cigarettes

Rejectors often compared the cost of ENDS to that of cigarettes and reported discontinuing ENDS because the perceived cost was too high. Rejectors perceived ENDS to be more expensive due to the cost of trying different devices and purchasing refills for cartridge systems.

*Male, Rejector: “Some of them [ENDS] are like 15 or 20 bucks. That’s way more than a pack of cigarettes.”*

*Female, Rejector: “Yeah, [the ENDS product] was more expensive than a cigarette, but I’d rather go cold turkey. I mean I’d rather go cold turkey than spend that much money for a product that didn’t even give me a little satisfaction. You know what I’m saying? I mean not even a little bit of satisfaction. I mean it’s just like just throwing away good money.”*

#### 3.6.4. Rejectors Were Concerned about the Health Risks of ENDS

Rejectors reported discontinuing use or having second thoughts about using ENDS because of concerns about the health risks of ENDS. When asked what risks they were concerned about, Rejectors cited devices exploding and hospitalized youths. They expressed concerns about the immediacy of these risks and indicated that they wanted proof that ENDS were safer than cigarettes before they would re-consider continuing ENDS use.

*Female, Rejector: “The e-cigarette, I thought it was the way out, but then with all of the health issues, I mean smoking tobacco, I mean come on. We know that can create health issues. But if I’m trying to stop smoking, then I’m going to take on something else that may bring on a sickness even quicker or cause some immediate health issues, right?”*

#### 3.6.5. Rejectors Were Not Interested in Flavored ENDS

Rejectors, like Never Users, were not enticed by ENDS flavors. Rejectors reported difficulty in finding a flavor that met their expectations. Several participants in these groups expressed the desire to find a flavor that mimicked their cigarettes.

*Female, Rejector: “It didn’t have any flavor in it, for me. It didn’t seem like—with cigarettes, you can kind of taste the tobacco, with the vapor it’s like you’re just blowing bland smoke out. You know?”*

### 3.7. Research Question 4: What Factors Facilitate a Successful Transition from Cigarette Smoking to Exclusive ENDS Use?

Unlike Rejectors and Dual Users, Switchers reported finding a product that was able to meet their needs. Most Switchers indicated that they had to try many different ENDS products before they found the “right” one. Switchers indicated that their ENDS products satisfied their cravings, were less harmful than cigarettes, and satisfied other use requirements.

#### 3.7.1. Switchers Found ENDS to Be a Satisfying Replacement for Cigarettes

Unlike Rejectors and Dual Users, Switchers asserted that once they found the “right” ENDS product, their ENDS product was able to satisfy their nicotine cravings while they were trying to quit smoking cigarettes. They found the throat hit and sensory experience of ENDS to be sufficiently similar to cigarettes. Some Switchers added that ENDS helped them reduce their nicotine consumption because of the customizable nicotine feature of some products.

*Female, Switcher: “I started with the vape and I noticed that it was nothing like before. I wasn’t having the withdraws, I wasn’t having the crazy—I was going for the vape more than I was going for my cigarettes, and I was like, “This is it.”*

#### 3.7.2. Switchers Perceived ENDS as Less Harmful to Their Health than Cigarettes

Unlike the other user groups, many Switchers asserted that ENDS carry fewer health risks than cigarettes. Some Switchers reported that they had even experienced health benefits from switching to ENDS such as breathing more easily.

*Male, Switcher: “Yeah, sometimes I’ll think about it. I’m like, ‘Maybe I shouldn’t be doing this [using ENDS]. Maybe this isn’t the best.’ But, then there’s other times that I don’t even think about it. I’m just like, ‘This is better than cigarettes. It’s a compromise.’ It’s unfortunate that I became addicted to cigarettes, but in a way I’m thankful for a somewhat healthy alternative that will eventually lead me to the path of completely quitting.”*

*Female, Switcher: “I’m almost a year in without smoking. I don’t miss it, but usually in the winter, me and my husband normally get the nastiest cold and I noticed that this year, neither one of us had anything. Our sinus infections are almost non-existent.”*

#### 3.7.3. After Some Trial and Error, Switchers Found an ENDS Product that Fit Their Needs

Although Switchers acknowledged that some ENDS products were not ideal because they were not the right size, broke often, or didn’t provide the right throat hit, Switchers said they were ultimately able to find the “right” product that fulfilled their needs. They reported trying a variety of products over weeks or months before finding their preferred product. With their preferred product, they reported experiencing fewer instances of it breaking, better user experience, and satisfaction with the look and feel of the product. These products were often customizable and had a long enough battery life to be convenient.

*Female, Switcher: “I have a box mod, and I love that it’s USB port that just—when I go to charge my phone at night, I charge my vape at night, as well. It’s good to go for the whole next day. I don’t have to worry about changing out batteries or anything like that.”*

#### 3.7.4. Switchers Found ENDS Flavors Appealing

Switchers found the variety of flavor options for ENDS appealing. Switchers discussed how the variety of flavors allows for a customizable use experience and a few even perceived that the flavors facilitated smoking cessation.

*Male, Switcher: “Yes, the flavors are a huge benefit. There’s so many different flavors out there. Pretty much anybody can find whatever they want—whatever suits them to help them quit smoking.”*

*Female, Switcher: “I’ve been vaping for nine years now. I tried smoking a cigarette a year after I started vaping and it totally gagged me. I couldn’t even fathom the taste. I was like, “I can’t believe I used to do this.” The flavors, yes. They’re talking about taking the flavors away. That’s crazy to me because I think that’s what, a lot of the people, helps them quit smoking is the flavors.”*

## 4. Discussion

The current research identified smokers’ perceptions of factors that affected their decisions of whether or not to try ENDS, and whether to continue or discontinue ENDS use after initiation (i.e., Never Users, Dual Users, and Rejectors, respectively). It also identified perceived factors that facilitated a successful transition from cigarettes to ENDS among former smokers who now use ENDS exclusively (i.e., Switchers).

The current investigation found that beliefs that ENDS can help facilitate smoking cessation [6,7] and perceptions that ENDS are more convenient than cigarettes (e.g., because they can be used in situations where cigarettes are not allowed or are not acceptable) [8,14,27] motivated smokers’ decisions to try ENDS. Never Users reported an interest in using ENDS to help them quit smoking or avoid smoke-free policies but had decided not to because they perceived the health risks of ENDS as equivalent to or greater than those of cigarettes. They also expressed concern about the costs of ENDS relative to cigarettes.

Both Dual Users and Rejectors reported frustration with ENDS products due to their perceived inability to satisfy cravings for cigarettes (including smoking rituals) and frequent device problems. Dual Users reported switching between ENDS and cigarettes depending on the situation because ENDS were convenient but unable to satisfy their cravings. Rejectors said this perceived inability to satisfy cravings caused them to discontinue ENDS use. Both Dual Users and Rejectors reported that the ENDS devices they used frequently break and need to be replaced. While Dual Users discussed managing this frustration by trying new ENDS products, Rejectors said that products breaking caused them to discontinue ENDS use and return to smoking. Additionally, Rejectors cited the perceived high cost of ENDS devices and refills as a reason for returning to cigarettes. Rejectors and Dual Users appear to experience the same frustrations with ENDS. However, in the case of Dual Users, either other benefits outweighed the frustrations or they were able to adjust (e.g., also smoking cigarettes), whereas, with Rejectors, the frustrations outweighed and perceived benefits of continued use.

Unlike Dual Users and Rejectors, smokers who successfully switched to ENDS said that they were able to find an ENDS product that satisfied their cravings. Switchers also reported experiencing ENDS device problems, but these problems motivated them to try new devices until they found one that met their needs. Switchers perceived ENDS to pose fewer health risks than cigarettes and often cited improvements in their own health after switching as evidence to support this perception. Switchers also asserted that the variety of flavors available for ENDS aided in their complete switching.

This investigation used a qualitative approach to build our understanding of the factors that affect smokers’ decisions to start and stop using ENDS. Although qualitative investigations are well suited to developing hypotheses, our sample is not intended to be representative and additional research is needed to determine the generalizability of findings. Cohort effects are possible, particularly around risk perceptions, which may have been affected by the outbreak of e-cigarette/vaping related lung injury which unfolded in the six months prior to data collection. Our conclusions about what caused smokers to abandon ENDS use or what helped them to switch products completely are necessarily retrospective on the part of our participants and thus subject to recall bias [28]. However, this study is strengthened by its unique focus on subgroups of smokers and former smokers based on their patterns of ENDS use.

Consistent with best practices in focus group research, we conducted three groups with each ENDS user type [23,24]. Each focus group included three to seven participants, which is modest by traditional focus group standards. However, four to six participants per group is considered to be the optimal size for online focus groups because it ensures that all participants are able to contribute to the discussion [29]. Indeed, participants engaged in rich discussion that was guided by the moderator, but with significant interaction between participants. Clear themes emerged across groups, consistently reaching saturation. Although our original recruitment profiles were approximately 50% male and 50% female, the individuals who ultimately participated were predominantly female (68.9%). This may reflect gender-based differences in flexibility to reschedule or comfort using online audio/visual technology, either of which could bias results. Additionally, demographics differed substantially between Switchers and other groups. Unlike other groups, smokers who successfully switched to ENDS were predominantly white (86.7%), compared to Never Users (27.3%), Rejectors (28.6%), and Dual Users (42.9%). Although this study is not designed to draw conclusions about differences in the relationship between race/ethnicity and successfully transitioning from cigarettes to ENDS, U.S. nationally representative data suggest that non-Hispanic Whites are more likely to use ENDS daily than members of racial and ethnic minorities [30]. Due to the small sample size and non-representative nature of our sample, we recommend replicating these findings in future quantitative research to ensure generalizability.

All participants were screened by telephone and determined to meet eligibility criteria for their assigned focus group at the time of screening, however, some participants reported ENDS use patterns that were inconsistent with the group’s inclusion criteria on the pre-focus group questionnaire. This discrepancy could be due to misreporting during the screening call, misreporting on the pre-focus group questionnaire, or changes in ENDS use behavior during the two to four weeks between the screening call and the focus group. Importantly, all participants described ENDS use patterns consistent with their assigned group during the discussion (i.e., Never Users indicated that they had never used ENDS regularly, Rejectors indicated that they no longer used ENDS regularly but owned a device, Dual Users discussed their current use of both cigarettes and ENDS, and Switchers indicated that they had transitioned from smoking cigarettes to using ENDS). One possible explanation for misreporting is that our inclusion criteria necessarily assigned categories to events that participants perceive as continuous, making it hard for participants to provide accurate information. For example, a Rejector may say that they no longer use ENDS during the screening call, but have purchased a single-use cig-a-like while traveling, with no intention of continued use. Although this would be inconsistent with our inclusion criteria, the participant would still be able to describe what they liked and disliked about ENDS and why they stopped using ENDS regularly. A Never User who had in fact used a friend’s product once could still describe why they had not made the effort to try using ENDS regularly. Never-the-less these inconsistencies do threaten our ability to draw conclusions, particularly among Never Users and Rejectors.

Findings from the current investigation should be replicated and expanded in future quantitative research. Switchers reported trying many products before finding the "right" one, often starting with cig-a-likes, switching to pod systems, and then switching to tank systems. Prospective longitudinal research is needed to build our understanding of what motivates Switchers to keep trying new products, what types of ENDS products smokers find most appealing, and what types of ENDS products most successfully facilitate transitions from smoking to ENDS use. Additional research is also needed to build our understanding of the contextual factors and individual differences that affect smokers’ transitions to ENDS. For example, smokers’ perceived convenience as an important reason for both trying and using ENDS. Finally, although non-users of ENDS perceived ENDS as having more health risks than cigarettes, Switchers perceived ENDS as having fewer health risks than cigarettes. Prospective longitudinal research is needed to understand whether this difference in risk perceptions are a cause or a consequence of switching products.

Several findings from this investigation point toward policy-related questions that should be answered in future research. First, ENDS users reported trying a variety of ENDS products (e.g., cig-a-likes, pod systems, tank systems) before finding one that satisfied their cravings. They reported heterogeneous preferences for other ENDS characteristics such as flavors and device types. Further research is needed to better understand the impact on policies that limit product diversity or (dis-)favor certain products, such as product standards, market authorization decisions, or taxation, on ENDS uptake, use patterns, and smoking outcomes. Second, developers of health communication campaigns should consider the impact of messaging about ENDS on the perceptions of addicted smokers. Smokers who did not use ENDS reported hesitance to use ENDS because they perceived the risk to be similar to or greater than that of cigarettes. Although it is important to prevent adolescents from using ENDS, messaging that discourages addicted smokers from switching products could have a negative impact on population-level health. Third, it is possible that extending cigarette prohibitions to ENDS could make smokers less likely to switch products. Participants reported that they tried ENDS because they could use them in places where cigarette smoking was not permitted, as well as reluctance to use ENDS because they were more expensive than cigarettes. Policy evaluation data are needed to assess the impact of extending policies intended to reduce combustible cigarette use (e.g., clean indoor air policies and taxation) on rates of product switching among addicted smokers.

## 5. Conclusions

Taken together, the current research suggests that a common set of factors including motivation to quit smoking, the convenience of using ENDS, and the availability of ENDS in a variety of appealing flavors influence smokers’ decisions to try ENDS. ENDS users who successfully quit smoking cigarettes reported trying several products before finding one that works for them. Contrastingly, ENDS users who returned to smoking reported being unable to find a product that satisfied their cravings and other needs. Although this study suggests several hypotheses, prospective longitudinal research is needed to more fully elucidate the processes that facilitate a successful transition from cigarettes to ENDS use. Meanwhile, we recommend that policymakers consider the potential impact of tobacco control policies on smokers’ motivation and ability to switch completely from cigarettes to ENDS.

## Figures and Tables

**Table 1:** Demographic information for study participants.

Characteristics	Mean (SD)/Frequency (*N*)				
	Never Users ^A^	Rejectors ^B^	Dual Users ^C^	Switchers ^D^	Total
Total Participants (*N*)	11	14	21	15	61
Atlanta, GA Group (*N*)	36.4% (4)	28.6% (4)	33.3% (7)	40.0% (6)	34.4% (21)
Baltimore, MD Group (*N*)	36.4% (4)	42.8% (6)	38.1% (8)	33.3% (5)	37.7% (23)
St. Louis, MO Group (*N*)	27.2% (3)	28.6% (4)	28.6% (6)	26.7% (4)	27.9% (17)
Age	52.3 (11.8)	50.1 (11.3)	44.1 (9.6)	39.7 (11.2)	45.9 (11.5)
Gender					
Male	27.3% (3)	21.4% (3)	28.6% (6)	46.7% (7)	31.1% (19)
Female	72.7% (8)	78.6% (11)	71.4% (15)	53.3% (8)	68.9% (42)
Sexual Orientation					
Heterosexual	100% (11)	64.3% (9)	85.7% (18)	93.3% (14)	85.2% (52)
Homosexual /Bisexual	0	35.7% (5)	14.3% (3)	6.7% (1)	14.8% (9)
Race					
American Indian or Alaska Native	0	0	0	0	0
Asian	0	0	0	0	0
Black or African American	72.7% (8)	71.4% (10)	52.4% (11)	0	47.5% (29)
Native Hawaiian or Pacific Islander	0	0	0	0	0
White	27.3% (3)	28.6% (4)	42.9% (9)	86.7% (13)	47.5% (29)
More than one race	0	0	4.8% (1)	13.3% (2)	4.9% (3)
Hispanic, Latinx, or of Spanish origin	0	0	14.3% (3)	0	4.9% (3)
Education					
12th grade or less, no diploma	9.1% (1)	0	4.8% (1)	0	3.3% (2)
High school graduate	27.3% (3)	14.3% (2)	14.3% (3)	26.7% (4)	19.7% (12)
GED ^E^ or equivalent	9.1% (1)	7.1% (1)	4.8% (1)	13.3% (2)	8.2% (5)
Some college, no degree	18.2% (2)	50% (7)	19% (4)	13.3% (2)	24.6% (15)
Associate degree	0	7.1% (1)	9.5% (2)	20% (3)	9.8% (6)
Bachelor’s degree	27.3% (3)	21.4% (3)	47.6% (10)	20% (3)	31.1% (19)
Master’s degree	9.1% (1)	0	0	6.7% (1)	3.3% (2)

^A^ People who reported having smoked 100 or more lifetime cigarettes, currently smoking cigarettes every day or some days, and never having used an electronic nicotine delivery systems (ENDS) device they own. ^B^ People who reported having smoked 100 or more lifetime cigarettes, currently smoking cigarettes every day or some days, having previously used an ENDS device they own every day or some days, and not currently using an ENDS device they own. ^C^ People who reported having smoked 100 or more lifetime cigarettes, currently smoking cigarettes every day or some days and currently using and ENDS device they own every day or some days. ^D^ People who reported having smoked 100 or more lifetime cigarettes, not currently smoking cigarettes, and currently using an ENDS device they own every day or some days. ^E^ The GED (formerly General Educational Development) test is a series of subject tests used in the United States as an alternative to a high school diploma to provide certification of high-school level academic knowledge.

**Table 2:** ENDS use patterns and product characteristics from the pre-focus group survey.

Use Patterns and Product Characteristics	Mean (SD)/Frequency (*N*)				
	Never Users ^A^	Rejectors ^B^	Dual Users ^C^	Switchers ^D^	Total
Participants (*N*)	11	14	21	15	61
Ever Use	18.2% (2)	100% (14)	100% (21)	100% (15)	85.2% (52)
Ever Use Quantity					
1 time	18.2% (2)	7.1% (1)	4.8% (1)	0	7.7% (4)
2–10 times	-	35.7% (5)	9.5% (2)	0	13.5% (7)
11–20 times	-	14.3% (2)	19% (4)	0	11.5% (6)
21–99 times	-	28.6% (4)	23.8% (5)	6.7% (1)	19.2% (10)
100 or more times	-	14.3% (2)	42.9% (9)	93.3% (14)	48.1% (25)
Last Use					
Earlier today	-	0	42.9% (9)	93.3% (14)	37.7% (23)
Not today but in the past 7 days	-	7.1% (1)	19% (4)	6.7% (1)	9.8% (6)
Not in the past 7 days but in the past 30 days	-	0	4.8% (1)	0	1.6% (1)
Not in the past 30 days but in the past 6 months	-	35.7% (5)	23.8% (5)	0	16.4% (10)
Not in the past 6 months but in the past year	9.1% (1)	21.4% (3)	9.5% (2)	0	9.8% (6)
1–4 years ago	-	35.7% (5)	0	0	8.2% (5)
5 or more years ago	9.1% (1)	0	0	0	1.6% (1)
Current Use					
Every day	-	0	42.9% (9)	93.3% (14)	37.7% (23)
Some days	-	21.4% (3)	52.4% (11)	6.7% (1)	24.6% (15)
Not at all	18.2% (2)	78.6% (11)	4.8% (1)	0	23% (14)
Product Characteristics					
Rechargeable	-	78.6% (11)	76.2% (16)	100% (15)	68.9% (42)
Tank system	-	35.7% (5)	42.9% (9)	80% (12)	42.6% (26)
Pod system	-	57.1% (8)	61.9% (13)	20% (3)	39.3% (24)
Refillable	-	50% (7)	42.9% (9)	86.7% (13)	47.5% (29)

^A^ People who reported having smoked 100 or more lifetime cigarettes, currently smoking cigarettes every day or some days, and never having used an ENDS device they own. ^B^ People who reported having smoked 100 or more lifetime cigarettes, currently smoking cigarettes every day or some days, having previously used an ENDS device they own every day or some days, and not currently using an ENDS device they own. ^C^ People who reported having smoked 100 or more lifetime cigarettes, currently smoking cigarettes every day or some days and currently using and ENDS device they own every day or some days. ^D^ People who reported having smoked 100 or more lifetime cigarettes, not currently smoking cigarettes, and currently using an ENDS device they own every day or some days.

**Table 3:** Coding themes.

Theme	Definition
Satisfaction	Perceived ability or inability of an Electronic Nicotine Delivery Systems (ENDS) product to satisfy cravings for cigarettes. This includes desires for nicotine, “hand-to-mouth” experience, comments about the sensory experience of inhaling the product, and comments about physiological responses to nicotine, such as perceptions of “throat hit” or “head rush”.
Smoking Cessation Beliefs	Beliefs that the ENDS product could, would, or did help participants quit smoking cigarettes. This also includes beliefs that the ENDS product could not, would not, or did not help participants quit smoking cigarettes.
Convenience	Perceptions that ENDS are convenient because they do not smell like cigarettes, they can be used in places where cigarette smoking is not allowed, or they can be used in social situations (or around certain people) when smoking is not acceptable. This can be due to rules, regulations, or social norms.
Risk Perceptions	Perceptions of the health risks associated with ENDS use. This includes e-cigarette or vaping associated lung injury (EVALI), long-term risks, such as cancer, and short-term risks, such as shortness of breath, popcorn lung, or exploding devices. These may be stated as health risks compared to cigarettes or anecdotal stories about health effects experienced while using ENDS.
Product Problems	Reports of difficulty finding a product that meets the participant’s needs (i.e., simple to use, fits in the user’s pocket or hand, easy to find replacements) or experiencing product-related issues (i.e., leaking, needing to charge too often, breaking frequently). This can also include positive product experiences, such as finding the “right” product that meets participants’ needs and expectations.
Cost Compared to Cigarettes	Perceptions of the cost of ENDS devices, refills, or replacements being cheaper or more expensive in comparison to cigarettes.
Flavor	Interest or lack of interest in the variety of flavors available for ENDS. This includes perceptions of the flavors being good or tasting like cigarettes as well as perceptions of the availability of adequate flavors.

**Table 4:** Summarization of main findings.

Research Question	Main Findings
1. Why do smokers decide to try using ENDS or not try using ENDS?	All groups were initially interested in ENDS because of smoking cessation beliefs.
	Perceived convenience made trying ENDS appealing for all groups.
	Risk perceptions may deter or encourage trying or re-trying ENDS.
	Cost relative to cigarettes deterred some from trying or re-trying ENDS and not others.
2. What factors drive dual users of cigarettes and ENDS to use both products rather than switching completely to ENDS?	Dual Users were motivated to continue ENDS use because of convenience.
	Dual Users found ENDS flavors appealing.
	ENDS were not satisfying enough to support switching for Dual Users.
	Product problems prevented Dual Users from switching completely to ENDS.
3. What factors drive smokers who have initiated ENDS use to discontinue and return to smoking cigarettes?	Rejectors did not find ENDS satisfying enough.
	Rejectors were frustrated by ENDS product problems.
	Rejectors perceived ENDS as more expensive than cigarettes.
	Rejectors were concerned about the health risks of ENDS.
4. What factors facilitate a successful transition from cigarette smoking to exclusive ENDS use?	Switchers found ENDS to be a satisfying replacement for cigarettes.
	Switchers perceived ENDS as less harmful to their health than cigarettes.
	After some trial and error, Switchers found an ENDS product that fit their needs.
	Switchers found ENDS flavors appealing.
